# Population pharmacokinetics and dose–response relationships of mitoxantrone in children with acute myeloid leukaemia

**DOI:** 10.1002/bcp.70436

**Published:** 2026-01-14

**Authors:** Andrew M. Brandon, Hinke Huisman‐Siebinga, Shelby Barnett, Paul Wetherell, Pamela Kearns, Brenda Gibson, Nicholas Heaney, Owen Smith, André Baruchel, Arnaud Petit, Andrew Moore, Kayode Ogungbenro, Alwin D. R. Huitema, Gareth J. Veal

**Affiliations:** ^1^ Centre For Cancer, Translational And Clinical Research Institute Newcastle University Newcastle upon Tyne UK; ^2^ Department of Pharmacy and Pharmacology The Netherlands Cancer Institute Amsterdam The Netherlands; ^3^ Cancer Research UK Clinical Trials Unit, School of Medical Sciences University of Birmingham Birmingham UK; ^4^ Royal Hospital for Children Glasgow UK; ^5^ Our Lady's Hospital for Sick Children Dublin Ireland; ^6^ Assistance Publique – Hôpitaux de Paris Hôpital Robert Debré Paris France; ^7^ Assistance Publique – Hôpitaux de Paris Hôpital Trousseau Paris France; ^8^ Queensland Children's Hospital Brisbane Queensland Australia; ^9^ Centre for Applied Pharmacokinetic Research, Division of Pharmacy and Optometry University of Manchester Manchester UK; ^10^ Department of Pharmacology Princess Máxima Center for Pediatric Oncology Utrecht The Netherlands; ^11^ Department of Clinical Pharmacy, University Medical Center Utrecht Utrecht University Utrecht The Netherlands

**Keywords:** acute myeloid leukaemia, mitoxantrone, paediatrics, pharmacokinetics

## Abstract

**Background:**

Information on mitoxantrone pharmacokinetics in children is lacking and reduced dosing regimens applied to infants are supported by limited scientific rationale. The current study characterized mitoxantrone pharmacokinetics in a childhood acute myeloid leukaemia patient population and provides a data‐informed assessment of dosing.

**Methods:**

A total of 282 plasma samples from 44 patients aged 0.9–17 years, receiving intravenous mitoxantrone at doses of 12 mg/m^2^/day or 0.4 mg/kg/day (patients <12 months, ≤10 kg or <0.5 m^2^), were analysed, and a population pharmacokinetic model was developed. Individual clearance (CL) values were used to calculate mitoxantrone area under the plasma concentration‐time curve (AUC) for each patient. Relationships among dosing regimen, pharmacokinetics and toxicity were assessed. Simulation of 1000 virtual patients, sampled from real covariate combinations, was used to investigate standardized patient dosing.

**Results:**

A two‐compartment model with fixed allometric scaling best described the data, with a final population estimated CL of 39.1 L/h (residual standard error 9.6%) observed for a patient weighing 27.5 kg. Infants receiving mg/kg dosing exhibited lower AUC values (192 ± 75 μg·h/L) than the mg/m^2^ group (317 ± 184 μg·h/L). Simulations showed that a standardized 12 mg/m^2^/day dosing regimen would likely result in comparable AUCs across all ages. No correlation was observed between mitoxantrone AUC and incidence of severe toxicity (Common Terminology Criteria for Adverse Events [CTCAE] grade 3/4) in this cohort.

**Conclusion:**

This study provides novel insights into the pharmacokinetics of mitoxantrone in children. Infant patients receiving body weight‐based dosing regimens may be at risk of suboptimal drug exposure, and many of these patients may tolerate higher mitoxantrone doses in line with older children. This trial was registered with the EU Clinical Trials Register (EudraCT number 2014‐005066‐30).

**What is already known about this subject**

- Mitoxantrone pharmacokinetic data in children are sparse, with limited clinical pharmacology information available to support evidence‐based dosing guidance.
- Chemotherapy dose reductions commonly applied to infants are supported by limited evidence and can lead to reduced drug exposure compared to standard dosing.

**What this study adds**

- This study improves understanding of mitoxantrone pharmacokinetics in children with acute myeloid leukaemia.
- Infants receiving reduced body weight‐based mitoxantrone doses risk suboptimal drug exposure and may benefit from an increased dosing regimen.

## 1 INTRODUCTION

Mitoxantrone is used in the treatment of multiple cancers, including leukaemia and non‐Hodgkin's lymphoma, as well as being used in the treatment of multiple sclerosis.^1^, ^2^ The use of mitoxantrone as part of induction chemotherapy for paediatric acute myeloid leukaemia (AML) is well established.^3^, ^4^ While AML in children and adolescents is rare, it is a significant cause of childhood cancer mortality, with a poor prognosis compared to other childhood leukaemias.^3^, ^5^, ^6^ While recent advances in paediatric AML treatment have improved patient outcomes, around 25–50% of patients will relapse.^3^, ^7^, ^8^

Mitoxantrone dosing regimens for AML are currently based on toxicity and efficacy data obtained from previous trials, with both adults and children typically being dosed at 10–12 mg/m^2^ via a short intravenous (IV) infusion. Reduced body weight‐based dosing is generally applied to younger children below a pre‐defined age, body weight or body surface area (BSA) cut‐off; however, such chemotherapy dose reductions in the youngest patients commonly lack standardization and are supported by limited scientific rationale.^9^, ^10^ The utility of body weight‐based dosing is widely employed in very young children, largely because of challenges in accurately determining BSA in smaller patients. It is unclear why the mg/kg dosing regimens proposed commonly incorporate an inherent dose reduction, as compared to mg/m^2^ dosing in older children, but this is likely related to concerns over drug tolerability in these very young patients or perceived changes in drug disposition related to differences in physiology. However, we have previously shown that such body weight‐based approaches to dosing can lead to the achievement of significantly lower and potentially sub‐therapeutic drug exposures for the commonly used anticancer drugs carboplatin and vincristine in neonate and infant patients,^11^, ^12^ with higher doses well tolerated in both cases.

The pharmacokinetics of mitoxantrone in adults is reasonably well documented, where it has been described by one, two and three‐compartment models,^13^, ^14^, ^15^, ^16^, ^17^ although reported pharmacokinetic parameter values vary widely. Mitoxantrone is primarily eliminated by biliary excretion as the parent drug or inactive metabolites.^15^, ^18^ Clearance (CL) of mitoxantrone is reduced in patients with hepatic impairment, with those with bilirubin concentrations of >3.4 mg/dL (58.1 μmol/L) shown to have an area under the plasma concentration–time curve (AUC) more than three times that of patients with normal hepatic function.^18^, ^19^ Despite the well‐characterized (albeit variable) pharmacokinetics of mitoxantrone in adults, information on its pharmacokinetics in children is lacking.^18^, ^20^ Previous studies of mitoxantrone pharmacokinetics in children have typically been multi‐drug studies involving relatively few patients.^21^, ^22^ In a review of cytotoxic drugs in neonates and infants, it was concluded that evidence‐based dosing guidance could not be provided for mitoxantrone because of a lack of published data.^17^

Dose‐dependent myelosuppression is a major, dose‐limiting toxicity of mitoxantrone^23^ and is observed alongside other common side effects, including cardiotoxicity^24^, ^25^ and mucositis.^1^ There is clearly a need for a more data‐informed assessment of mitoxantrone dosing in childhood cancer. Here, we present the development of a mitoxantrone population pharmacokinetic (popPK) model in a childhood AML population and explore relationships among dosing regimen, pharmacokinetics and toxicity, with the aim to inform better prescribing practice for this patient group.

## 2 PATIENTS AND METHODS

### 2.1 Patients and treatment

Data were generated from a phase III clinical trial in children with AML (ISRCTN12389567). The study protocol was approved by the Health and Care Research Wales Research Ethics Service (REC 15/WA/0316), and the trial was registered with the Medicines and Healthcare Products Regulatory Agency. Written informed consent was obtained from patients or parents/guardians as appropriate. Inclusion criteria for patients included newly diagnosed AML, high risk myelodysplastic syndrome, isolated myeloid sarcoma and age under 18 years at trial entry. Patient characteristics, baseline toxicity and additional clinical parameters were collected prior to mitoxantrone treatment. This included patient age, sex, height, body weight, BSA and concomitant therapies. Haematology and biochemistry measurements consisted of baseline haemoglobin, white blood cell and platelet counts, alanine aminotransferase (ALT), bilirubin, serum albumin, creatinine and estimated glomerular filtration rate (eGFR).

Mitoxantrone was administered by IV infusion over 1 h, once per day for up to 4 days, at a dose of 12 mg/m^2^/day. For infants <12 months old, weighing ≤10 kg or with a BSA of <0.5 m^2^, mitoxantrone was administered at a dose of 0.4 mg/kg/day. Following treatment, toxicity was monitored, and Common Terminology Criteria for Adverse Events (CTCAE) grade 3 and 4 toxicities recorded.

### 2.2 Sample collection and analysis

Blood samples (2 mL) were collected in ethylenediaminetetraacetic acid (EDTA) tubes prior to mitoxantrone administration, immediately after the end of infusion on day 1, at 0.5, 1, 2 and 6 h post‐infusion on day 1, immediately before drug infusion on day 2, and 48 and 72 h post‐end of final day infusion. Actual sample times were recorded for each blood sample collected for pharmacokinetic analysis, while not all samples were taken from all patients, such as in very young patients. Following collection, blood samples were immediately centrifuged at 1500 × *g* for 5 min at 4°C. Plasma samples were transferred to clean labelled tubes and stored at −20°C prior to analysis by high‐performance liquid chromatography–photo diode array (HPLC‐PDA) assay. The bioanalytical assay was fully validated in accordance with existing European Medicines Agency and U.S. Food and Drug Administration guidelines.^26^, ^27^

For extraction, the sample, quality control or standard (100 μL) was added to 3% (*v/v*) 5‐sulfosalicylic acid in deionized water (50 μL). Acetonitrile (150 μL) was added while mixing, and samples were centrifuged at 20800 × *g* for 5 min at 4°C. Supernatant (200 μL) was removed and evaporated to dryness under nitrogen at 30°C. Samples were reconstituted in 80:20 (*v/v*) aqueous 10 mM sodium phosphate buffer (pH 2.3):acetonitrile containing 0.1% (*v/v*) triethylamine. Samples were mixed and centrifuged at 20800 × *g* for 5 min at 4°C, and supernatants were transferred to clean vials for analysis.

HPLC‐PDA analysis was achieved using either an Agilent 1260 Infinity HPLC coupled to an Agilent Infinity 1100 PDA detector (Agilent Technologies, Cheadle, UK) or a Waters Alliance 2695 HPLC coupled to a Waters 2487 PDA detector (Waters Corporation, Wilmslow, UK), with Chromeleon software (version 7; ThermoScientific, Loughborough, UK) used for chromatogram interpretation. Samples (100 μL) were injected onto a HyperClone BDS C_18_ (250 × 4.6 mm, 5 μm) column (Phenomenex Ltd., Macclesfield, UK) with a SecurityGuard C_18_ (4 × 3 mm) guard column (Phenomenex Ltd.). Mobile phase was isocratic, comprising 80:20 (*v/v*) aqueous 10 mM sodium phosphate buffer (pH 2.3):acetonitrile with 0.1% (*v/v*) triethylamine. The run time was 7 min, and mitoxantrone was detected at a wavelength of 610 nm. Linear calibration range of the assay was 5–1000 ng mL^−1^, and the lower limit of quantification (LLOQ) was 5 ng mL^−1^. Inter‐ and intra‐assay precision and accuracy were tested across the calibration range, with concentrations within 15% of nominal values in all cases.

### 2.3 Pharmacokinetic analysis

A popPK model was developed using NONMEM version 7.5 (ICON Plc, Dublin, Ireland), with Finch Studio version 1.6 (Enhanced Pharmacodynamics LLC, Buffalo, NY, USA), Perl‐speaks‐NONMEM (PsN) (Uppsala Pharmacometrics, Uppsala, Sweden) and R version 4.5.1 used for model management and data analysis. A total of 313 samples were initially obtained from 44 patients. Because of the extent of late time‐point sampling, a high proportion (40.6%) of data in the original dataset were below the lower limit of quantification (BLLOQ). Some samples showed artificially high mitoxantrone concentrations directly after administration that were clearly skewed as compared to the majority of collected data, and these were excluded from analysis. The effect of re‐including these data were tested on the final model. A total of 282 concentration measurements (with 45.7% BLLOQ) from 145 dosing events were used for model development.

Two‐ and three‐compartment models were tested, parameterized as systemic clearance (CL), central and peripheral volumes of distribution, and inter‐compartmental clearances. Initially, first‐order conditional estimation with interaction (FOCE‐I) was used, and later stochastic approximation expectation maximization (SAEM). With SAEM estimation, a separate importance sampling (IMP) evaluation‐only estimation step was included to generate the objective function value (OFV, equal to minus two times the log likelihood) for the model. Both proportional and combined (additive and proportional) residual error models were tested. Early model development involved imputing BLLOQ concentrations as half the LLOQ (i.e. 2.5 ng mL^−1^). Later, the likelihood‐based M3 method was utilized, with all key runs performed using this method.^28^, ^29^

As the patient cohort were children, allometric scaling of body weight was implemented in the base model,^30^ with parameters scaled to the dataset median body weight of 27.5 kg (Equation 1).

(1) $$\mathrm{TVP}=\theta \times {\left(\frac{{\mathrm{WT}}_{i}}{{\mathrm{WT}}_{\mathrm{med}}}\right)}^{k}$$

Equation 1 Allometric scaling. *Where TVP is the typical value of the pharmacokinetic parameter; θ is the population value of the pharmacokinetic parameter; WT*_*i*_ *is the individual body weight (kg); WT*_*med*_ *is the population median body weight (kg); k is the exponent applied for allometric scaling*.

Inter‐individual variability (IIV) was then tested systematically on all pharmacokinetic parameters, which was then revisited later in model development (Equation 2). Covariance of random effects was also investigated.

(2) $$P_{i}=\mathrm{TVP}\times e^{\eta_{i}}$$

Equation 2 Inter‐individual variability. *Where P*_*i*_ *is the individual pharmacokinetic parameter value; η*_*i*_ *is the random effect (inter‐individual variability) for an individual patient and is assumed to be normally distributed with a mean of zero and a variance of ω^2^*.

Covariate effects tested were based on physiological relevance and on plots of covariates *vs*. random effects (*η*_*i*_). Covariate models tested included fixing (0.75 for clearances and 1 for volumes) and estimating allometric exponents, BSA (power model scaled to population median) instead of allometry, age (linear, power and maturation function [sigmoidal E_max_/Hill equation^31^]) and liver function (bilirubin and ALT as linear and power models). Each covariate effect was tested univariately against physiologically relevant combinations of parameters and were considered significant (*p* < 0.05) if they produced an OFV reduction of ≥3.84.

Models were evaluated using a combination of OFV, informed assessment of final parameter estimates, goodness‐of‐fit (GOF) plots and visual predictive checks (VPCs). Normalized prediction distribution errors (NPDE) evaluation was extended to BLLOQ data as described previously.^32^ Parameter precision was obtained by the $COVARIANCE option in NONMEM and, if possible, by sampling importance resampling.^33^

### 2.4 Mitoxantrone exposure and clinical toxicity

Relationships among dosing regimen, pharmacokinetics and exposure were assessed graphically and numerically. Maximum a posteriori Bayesian estimates of individual CL values were obtained for each patient (POSTHOC option in NONMEM) and used to calculate the AUC using dose and individual CL.

Categorical clinical toxicity data were available for each patient, with severe (CTCAE grade 3–4) toxicity observations recorded for at least 28 days post‐treatment. Correlation of AUC and CL with the incidence of severe toxicity was assessed by visual comparison. Incidence of severe toxicity was tested for significance between dosing groups and with or without the co‐administration of cytarabine by Mann–Whitney *U* test, a non‐parametric test for independent groups that does not assume data normality, with significance accepted as *p* < 0.05.

### 2.5 Simulation

Simulations were performed using the R ‘mrgsolve’ package^34^ to further assess AUC and to investigate alternative dosing regimens. Real covariate combinations were drawn from patient cohorts comparable to the model development dataset (442 patients aged 1 day to 18 years) and were randomly sampled with replacement to provide 1000 patients (with 500 eligible for mg/kg dosing and 500 eligible for mg/m^2^ dosing).

Mitoxantrone concentrations and CL values were simulated by dosing according to the recommended schedules, with dose and CL subsequently used to calculate AUC values. Simulated AUC values were compared between the original dosing regimen (1‐h IV infusion of 0.4 mg/kg if <12 months, ≤10 kg or <0.5 m^2^, otherwise 12 mg/m^2^) and a standardized dosing regimen of 12 mg/m^2^ (1‐h IV) for all patients.

### 2.6 Nomenclature of targets and ligands

Key protein targets and ligands in this article are hyperlinked to corresponding entries in http://www.guidetopharmacology.org, and are permanently archived in the Concise Guide to PHARMACOLOGY 2021/2022.^35^

## 3 RESULTS

### 3.1 Patient characteristics

Forty‐four paediatric patients were included in this study, with their characteristics summarized in Table 1. Patients were issued either the standard BSA‐based dosing regimen (mg/m^2^; *n* = 41) or a reduced body weight‐based dosing regimen (mg/kg; *n* = 3). Serum creatinine, ALT and serum albumin data were not available for two patients each, so were imputed as the population medians of 36.0 μmol/L, 42.5 U/L, and 35.5 g/L, respectively. Height information was not available for 21 patients; these were estimated using UK‐WHO growth charts based on patient sex, age and weight. Missing eGFR data (nineteen patients) was estimated using serum creatinine concentration along with known or estimated height using the revised Schwartz equation.^36^ All patients received non‐chemotherapeutic concomitant medications for management of symptoms and side effects. Sixteen (36.4%) patients received concomitant cytarabine (30–180 mg IV) within 7 days of commencing mitoxantrone treatment.

**TABLE 1:** Patient characteristics.

Dosing regimen	All patients	mg/m^2^ (*n* = 41)	mg/kg (*n* = 3)
Characteristic	Mean ± SD (median, range)		
Age (years)	9.6 ± 4.4 (9.7, 0.9–17.0)	10.2 ± 3.8 (9.8, 1.6–17.0)	1.1 ± 0.3 (0.9, 0.9–1.4)
Body weight (kg)	33.3 ± 16.8 (27.5, 9.5–69.5)	35.0 ± 16.1 (29.5, 13.0–69.5)	9.7 ± 0.2 (9.7, 9.5–9.9)
Body surface area (m^2^)	1.09 ± 0.38 (0.98, 0.42–1.84)	1.14 ± 0.36 (1.06, 0.59–1.84)	0.46 ± 0.03 (0.47, 0.42–0.48)
Height (cm)	134.8 ± 27.9 (134.2, 71.0–179.6)	139.1 ± 23.4 (141.5, 82.0–179.6)	74.8 ± 3.8 (75.0, 71.0–78.5)
Serum creatinine (μmol/L)	37.7 ± 13.7 (36.0, 14–74)	39.1 ± 13.0 (36, 17–74)	17.7 ± 3.2 (19, 14–20)
eGFR (mL/min/1.73 m^2^)	138.1 ± 28.6 (133.7, 88.7–229.7)	136.6 ± 28.2 (131.6, 88.7–229.7)	158.6 ± 32.4 (143.5, 136.6–195.8)
Alanine aminotransferase (U/L)	53.0 ± 47.1 (42.5, 6–184)	50.0 ± 45.8 (40, 6–184)	93.8 ± 56.3 (85, 43–154)
Bilirubin (μmol/L)	7.4 ± 4.2 (6.0, 3–19)	7.7 ± 4.2 (6, 3–19)	4.3 ± 1.5 (4, 3–6)
Serum albumin (g/L)	33.8 ± 6.5 (35.5, 4–43)	33.5 ± 6.7 (35.5, 4–43)	36.5 ± 3.1 (35.5, 34–40)
Dose (mg)	12.9 ± 4.8 (12, 3.6–23)	13.6 ± 4.2 (12, 7–23)	3.8 ± 0.1 (3.8, 3.6–3.82)
Infusion duration (h)	1.22 ± 0.31 (1.08, 0.5–2.5)	1.25 ± 0.30 (1.17, 0.92–2.50)	1.16 ± 0.24 (1.05, 1.00–1.43)
Number of samples per patient	7.1 ± 1.3 (8, 3–8)	7.1 ± 1.3 (8, 3–8)	6.7 ± 0.6 (7, 6–7)
	Number (%)		
Sex	M 27 (61.4%); F 17 (38.6%)	M 26 (63.4%); F 15 (36.6%)	M 1 (33.3%); F 2 (66.7%)
Diagnosis	AML 40 (90.9%); IMS 3 (6.8%); HRMS 1 (2.3%)	AML 37 (90.2%); IMS 3 (7.3%); HRMS 1 (2.4%)	AML 3 (100%)
FAB classification	M0 1 (2.3%); M1 1 (2.3%); M2 10 (22.7%); M4 8 (18.2%); M5 9 (20.5%); M7 2 (4.5%); Unavailable 13 (29.5%)	M0 1 (2.4%); M1 1 (2.4%); M2 9 (22.0%); M4 8 (19.5%); M5 9 (22.0%); Unavailable 13 (31.7%)	M2 1 (33.3%); M7 2 (66.7%)

Abbreviations: AML, acute myeloid leukaemia; eGFR, estimated glomerular filtration rate; F, female; FAB, French–American–British; HRMS, high risk myelodysplastic syndrome; IMS, isolated myeloid sarcoma; M, male; SD, standard deviation.

### 3.2 Population pharmacokinetics

**FIGURE 1:**
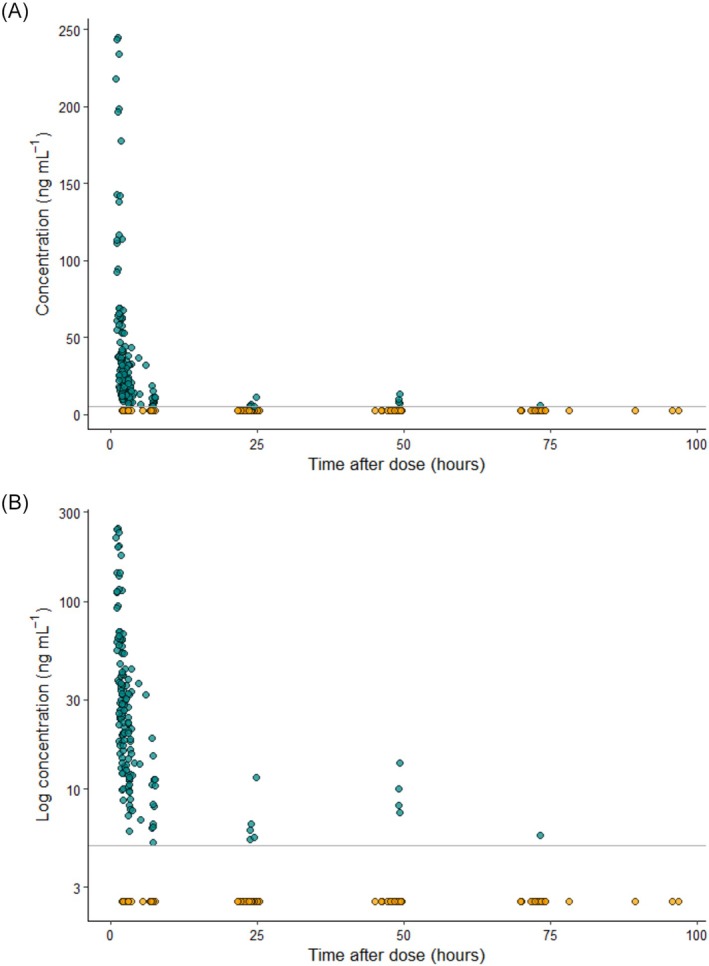
Observed mitoxantrone plasma concentrations *vs*. time after dose with (A) normal *Y*‐axis and (B) log‐transformed *Y*‐axis. The solid grey line represents the lower limit of quantification (5 ng/mL), while points below the lower limit of quantification are coloured orange and were inputted at 2.5 ng/mL (LLOQ/2).

Plots of observed plasma concentrations *vs*. time after dose (TAD) are provided in Figure 1. Two‐ and three‐compartment models showed comparable performance. Precedence was given to the two‐compartment versions, as these were simpler models yet still described the data adequately. Introduction of the M3 method for handling BLLOQ data resulted in an improved model fit, with GOF plots showing notably improved fit at late time points. As the dataset comprised of children, allometric scaling of body weight was included in the base model, using fixed exponents of 0.75 and 1.0 for clearances and volumes, respectively. No other clinically relevant covariate effect was found to be significant. Including BSA as a covariate instead of allometric scaling did not improve the model, nor did estimating the allometric scaling exponents. V1 was fixed to an existing literature value^22^; in later development, estimating V1 did not produce any notable change in final estimates but reduced model stability between repeated runs. Plots showing the correlation and distribution of covariates are provided in the Supporting Information, Section S1.

The final model comprised two compartments, parameterized as CL, V1, Q and V2, with IIV on CL and V2. V1 was fixed to 23.2 L^22^ to improve model stability. Residual unexplained variability was included as a combined (additive + proportional) error model, with the additive error fixed to 2.5 ng mL^−1^, reflecting half the assay LLOQ. The artificially high concentration samples taken exactly at the end of infusion, when re‐included, were poorly estimated according to GOF plots and produced a substantial increase in estimated proportional error, so remained excluded in the final model. Final model parameters are provided in Table 2.

**TABLE 2:** Mitoxantrone pharmacokinetic parameter estimates for the final popPK model.

Parameter	Estimate	RSE (%)
CL (L/h)	39.1	9.61
V1 (L)	23.2 fixed	‐
Q (L/h)	27.6	25.0
V2 (L)	85.9	22.6
WT effect on CL Q	0.75 fixed	‐
WT effect on V1 V2	1 fixed	‐
IIV CL (CV% [shrinkage %])	64.0 (10.65)	26.2
Covariance of IIV CL ~ V2	−0.426	43.0
IIV V2 (CV% [shrinkage %])	133 (34.11)	64.4
Proportional error	0.382	8.91
Additive error	2.5 fixed	‐

Abbreviations: CL, clearance; IIV, inter‐individual variability; Q, inter‐compartmental clearance; RSE, relative standard error obtained from the NONMEM $COV option; V1, volume of distribution, central compartment; V2, volume of distribution, peripheral compartment; WT, body weight. Parameter values are based on the median body weight of 27.5 kg. IIV RSEs are based on the standard errors of the estimated variances.

**FIGURE 2:**
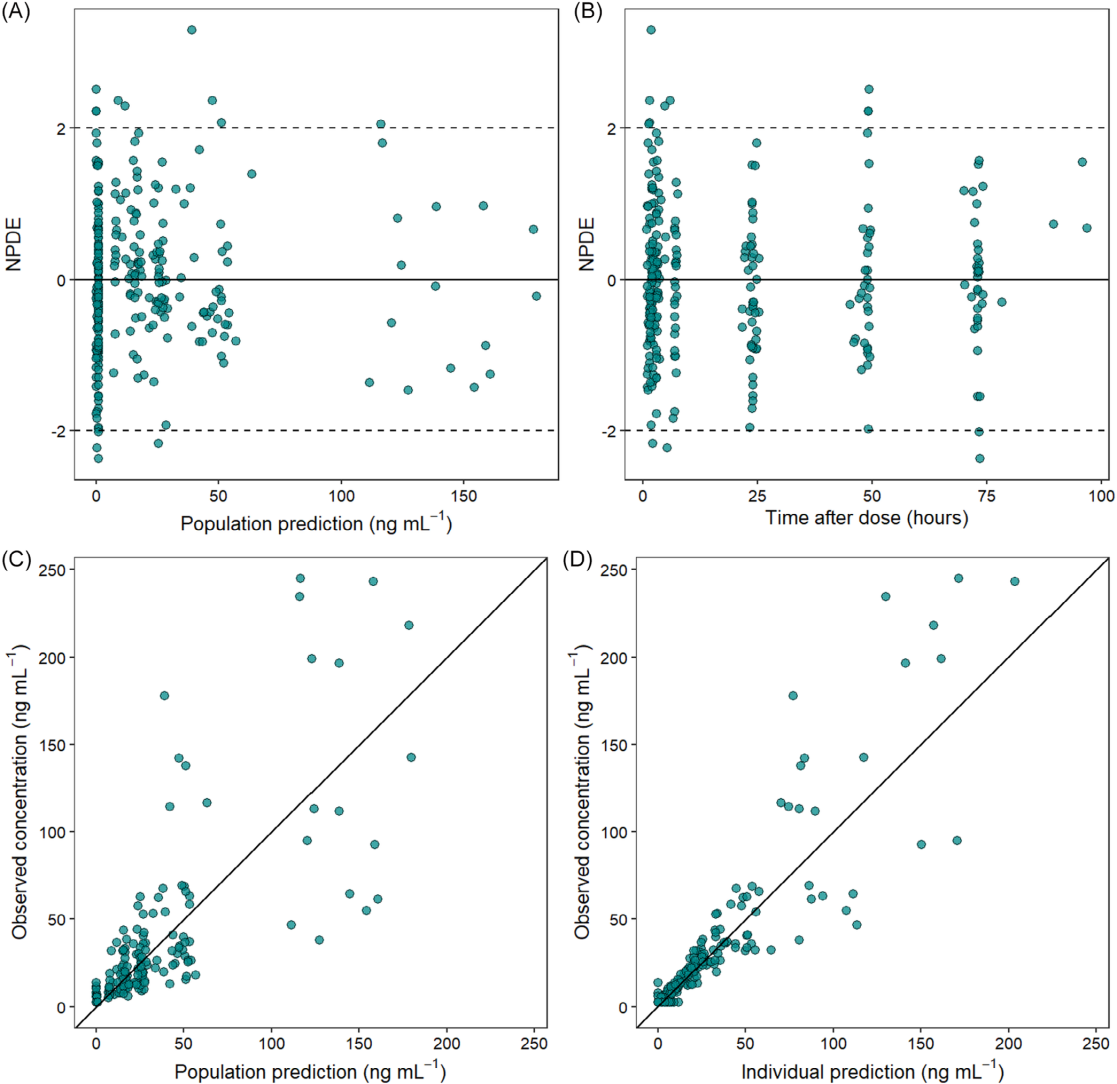
Goodness‐of‐fit plots for the final model. (A) Normalized prediction distribution errors (NPDE) *vs*. population predictions; (B) NPDE *vs*. time after dose (TAD); (C) observed concentrations *vs*. population predictions; and (D) observed concentrations *vs*. individual predictions.

**FIGURE 3:**
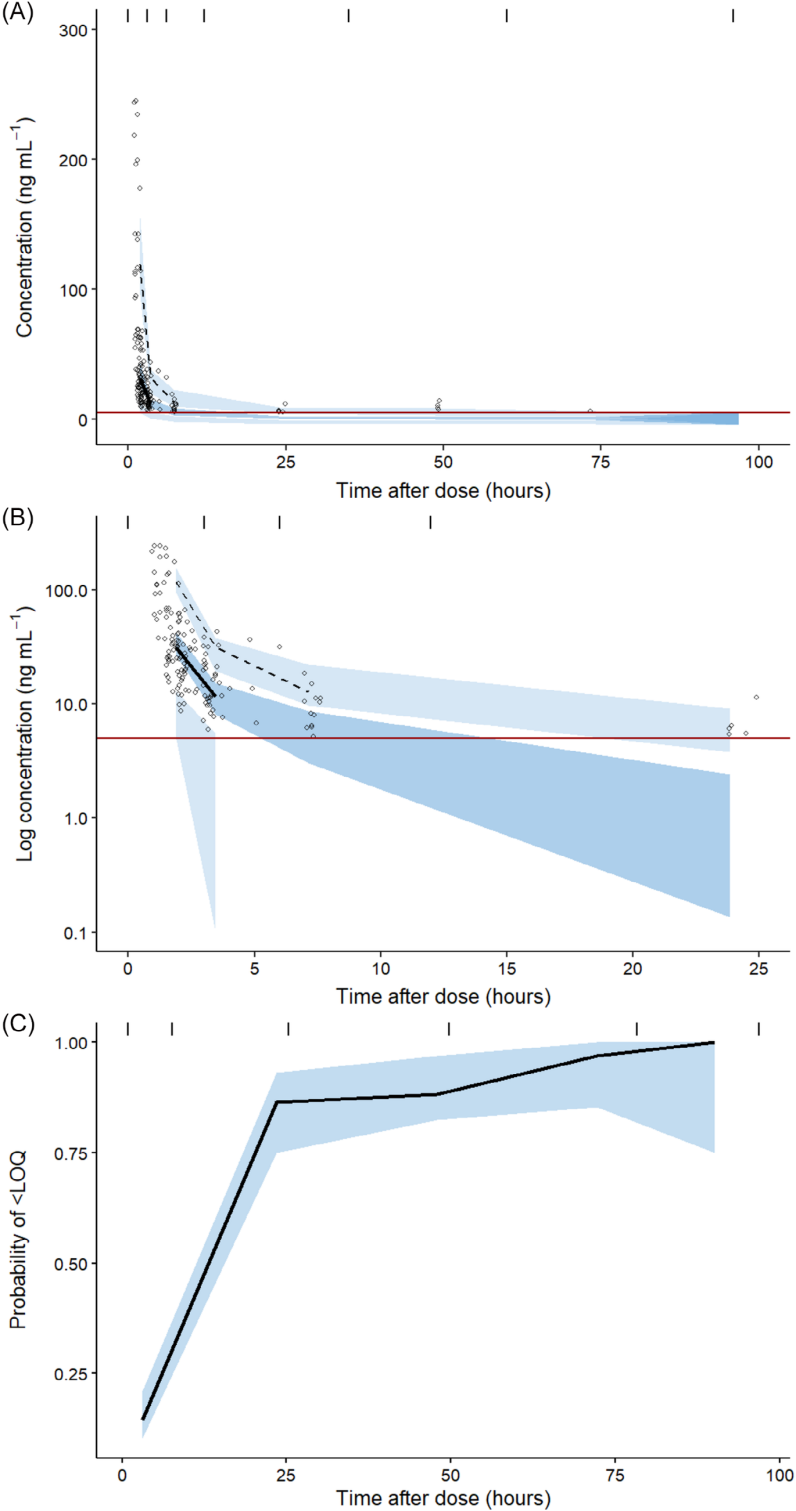
Visual predictive checks for the final model. (A) Linear scale. (B) Log concentration scale showing time 0–25 h. (C) Probability of measurements being below the lower limit of quantification over time. Individual data points represent observed data, with solid black lines being the 50th percentile of observations, and the upper dashed line being the 97.5th percentile. The lower dashed line (2.5th percentile) is not shown as it falls below the LLOQ (solid red line); instead, the probability plot (C) is used. Shaded areas represent the equivalent percentiles of simulated data. VPCs are based on 1000 simulated samples.

The final model described the data well, according to GOF plots (Figure 2) and VPCs (Figure 3). The majority of NPDE values (95.4%) were within the −2 to 2 range. The final model code is provided in the Supporting Information, Section S2. Additional VPC plots are provided in the Supporting Information, Section S3.

### 3.3 Comparing exposure, dosing regimen and toxicity

Estimated mitoxantrone AUC across all patients ranged 309 ± 181 μg·h/L. Patients receiving the mg/kg (n = 3) dosing regimen obtained lower AUCs compared to the mg/m^2^ (n = 41) group (mg/m^2^ = 317 ± 184 μg·h/L; mg/kg = 192 ± 75 μg·h/L), indicating potential under‐dosing of the mg/kg group.

Severe clinical toxicity was frequently reported in this cohort, with 47 distinct toxicity categories observed among all patients, and six being observed in over 20% of patients (febrile neutropenia [27.3%], decreased platelets [27.3%], decreased neutrophils [25.0%], anaemia [22.7%], decreased lymphocytes [22.7%] and decreased white blood cells [22.7%]). All other toxicities were reported in <7% of patients. Further detail on the occurrence of severe toxicity in patients is provided in the Supporting Information, Section S4. Despite the frequency of treatment‐related toxicity, there was no correlation observed between severe (grade 3–4) toxicity, AUC or CL in this patient group. There was also no statistically significant effect of dosing regimen or the co‐administration of cytarabine on severe myelosuppression‐related toxicity (*p* < 0.05). Further exploration of the relationships among toxicity, dosing regimen, AUC and CL is provided in the Supporting Information, Section S4.

### 3.4 Simulation

**FIGURE 4:**
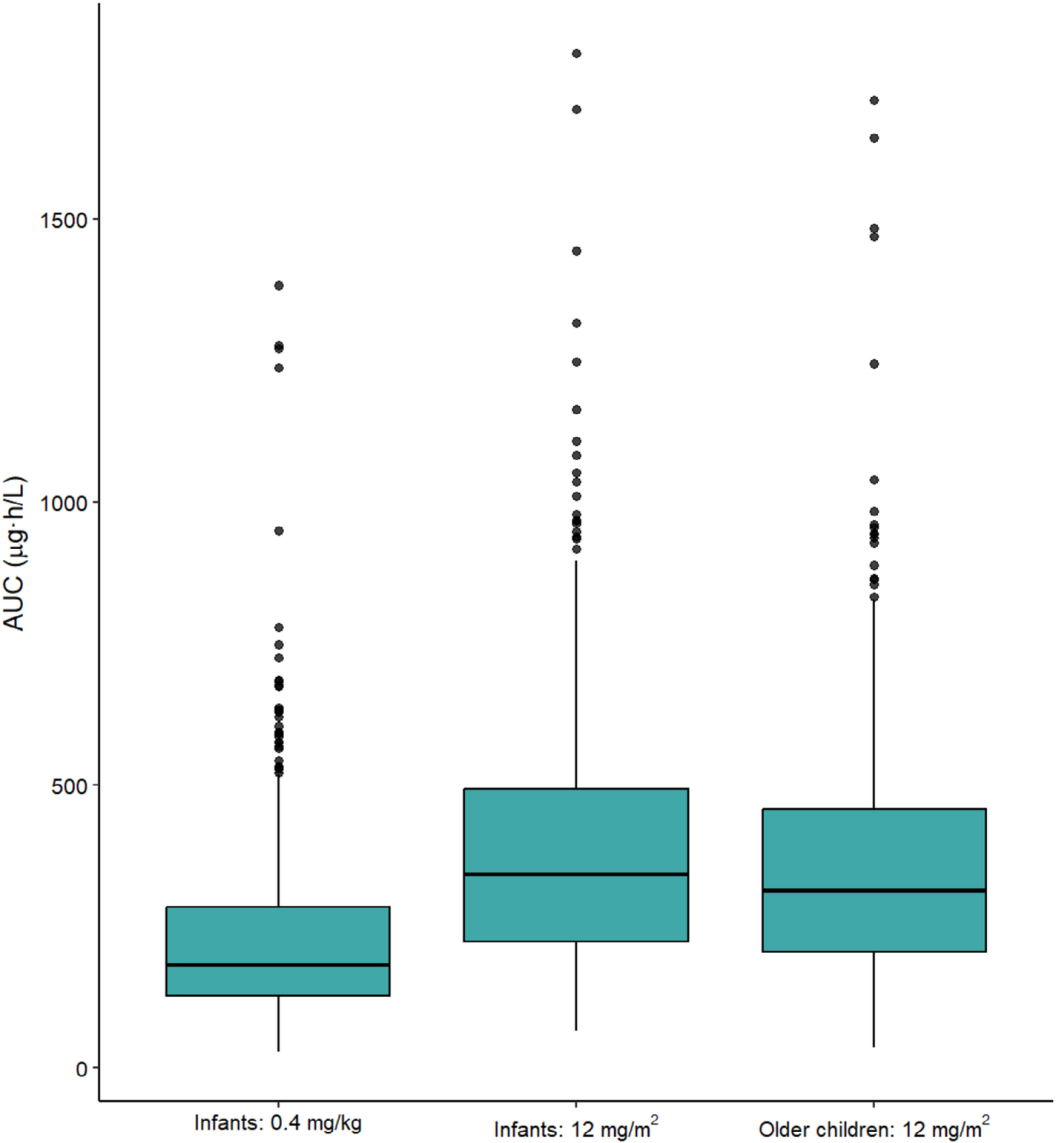
Simulated mitoxantrone area under the plasma concentration–time curve (AUC) for simulated patients eligible for 0.4 mg/kg dosing (<12 months, ≤10 kg or BSA < 0.5 m^2^), the same patients if administered 12 mg/m^2^ and patients eligible for 12 mg/m^2^ dosing (>12 months, ≥10 kg or BSA > 0.5 m^2^). Each simulation was based on a single 1‐h intravenous infusion, with 500 individuals in each group.

AUCs generated for 1000 virtual patients were comparable to those obtained from the model training dataset: with the original dosing (both body weight and BSA‐based dosing), simulated AUC across all patients ranged 299 ± 212 μg·h/L. Simulated patients receiving 0.4 mg/kg or 12 mg/m^2^ doses produced AUCs ranging 234 ± 171 μg·h/L and 388 ± 234 μg·h/L, respectively. When dosed at 12 mg/m^2^, AUCs of those previously dosed at 0.4 mg/kg increased to 365 ± 229 μg·h/L. AUC ranges for each dosing group are depicted in Figure 4.

## 4 DISCUSSION

Despite the long‐standing use of mitoxantrone in the treatment of paediatric AML and other cancers,^3^, ^4^ available data on its pharmacokinetics in paediatrics are scarce.^18^, ^20^, ^21^, ^22^ Current dosing approaches are based on limited scientific evidence, and there is a need for more data to support current mitoxantrone dosing regimens used in a childhood cancer setting.^9^, ^10^, ^17^ The current study aimed to improve our understanding of mitoxantrone pharmacokinetics in children, assess the suitability of reduced dosing regimens in the youngest patients and to explore relationships among mitoxantrone pharmacokinetics, exposure and toxicity in this patient cohort.

Final parameter estimates in this study were comparable to previously published findings from paediatric studies. O'Brien et al. (2010)^22^ described mitoxantrone pharmacokinetics in 21 subjects aged 2.3–20.3 years using a linear two‐compartment model. Patients were administered mitoxantrone at a dose of 10 mg/m^2^. Following a single IV dose, reported pharmacokinetic parameters were CL 16.4 L/h, V1 23.2 L, V2 102 L, and Q 8.13 L/h^22^, with lower CL and inter‐compartmental CL than observed in the present study (CL 39.1 L/h; Q 27.6 L/h). Lacayo et al. (2002)^21^ reported mitoxantrone pharmacokinetics in 12 children administered mitoxantrone alone, who were a subset of a patient cohort ranging 0.7–17 years and 8.4–134 kg. Patients were dosed IV at 10 mg/m^2^, and using a linear three‐compartment model, reported BSA‐scaled pharmacokinetic parameters (mean ± SD) were: CL 57 ± 19 L/h/m^2^; V1 40 ± 26 L/m^2^; AUC 194 ± 86 μg·h/L^21^, showing higher CL and V1, and lower AUC on average compared to this study. Mitoxantrone CL values reported by others were within the range of those reported in the present work, and differences in observed PK may in part be because of differences in study size. In the presented model, V1 was fixed to the value reported by O'Brien et al,^22^ which was based on a comparable patient population to ours (2.3–20.3 *vs*. 0.9–17 years; mean BSA 1.3 *vs*. 1.09 m^2^).

In adults, reported mitoxantrone PK is variable, with CL ranging from 11 to 37 L/h/m^2^ and total volume of distribution ranging from 322 to 2248 L/m^2^.^13^, ^14^, ^15^, ^19^, ^37^, ^38^, ^39^ CL in the present study is equivalent to the adult range, while volume of distribution was lower on average, which was also the case for other published paediatric data.^21^, ^22^ Across all studies, mitoxantrone volume of distribution is seen to be highly variable, as are the optimum number of model compartments reported. This has previously been, in part, attributed to the varying sampling density of late time points between studies.^13^ Data used for the present study included richly sampled late time points. Because of the high proportion of BLLOQ data (>40%), simply excluding these points or imputing them at half the LLOQ value would introduce bias to the results.^29^ Thus, the likelihood‐based approach was required.

Infant patients on a reduced mg/kg dosing regimen in the current study, although limited in number, had comparable CL to older children receiving mg/m^2^ dosing. These patients therefore exhibited lower exposure (AUC) because of the reduced dose received, indicating potential under‐dosing. These three patients were aged 10–16 months and weighed 9.5–9.9 kg, placing them close to the cut‐off for body weight‐based dosing, and so may not be reflective of all patients eligible for the reduced dosing regimen. If the patients receiving body weight‐based dosing in the current study had received 12 mg/m^2^ mitoxantrone, predicted AUC values would increase from 192 ± 75 μg·h/L to 278 ± 95 μg·h/L, more in line with exposures observed in the mg/m^2^ dosing group (317 ± 184 μg·h/L). Simulations based on a large virtual patient dataset confirmed these findings, where standardized 12 mg/m^2^ dosing was found to produce comparable AUCs across paediatric patients ranging from 1 day to 18 years in age. Further investigation is required to confirm these findings in a larger real‐world population of infant patients administrated mitoxantrone for the treatment of AML. However, the current findings support previous studies calling for updated, evidence‐based chemotherapy dosing guidelines for neonates and infants.^9^, ^10^, ^11^ While no clear conclusions can be drawn for mitoxantrone based on the results described, because of the small number of patients studied and lack of correlative data between exposure and clinical response/toxicity, the suggestion that body weight‐based dosing may be associated with the achievement of lower drug exposures clearly warrants further study. The current findings are consistent with those from similar studies, showing that infants receiving vincristine and carboplatin also commonly exhibit lower drug exposures than older patients receiving BSA‐based dosing. In these previously published studies, increased drug doses in the very young patients (to dose levels that equate to BSA‐based dosing) were well tolerated.^11^, ^12^, ^40^

The most frequently observed severe toxicities in this cohort were related to myelosuppression, a common major side effect of mitoxantrone treatment.^23^ Despite its frequency, the incidence of severe toxicity was not correlated with dosing regimen, AUC or CL. Similarly, Lacayo et al.^21^ found no difference in mean mitoxantrone AUC between patients with and without severe (grade 3/4) toxicity. The cardiotoxic effects of mitoxantrone^24^, ^25^, ^41^ are linked to cumulative dose and often show delayed onset, but the current study was limited to short‐term toxicity data. However, there is currently no evidence to suggest that infants require reduced mitoxantrone drug exposure in relation to cardiotoxicity. The available data did not extend beyond the dosing events used in modelling and so total cumulative dose for each patient in this study is unknown, and its correlation with myelosuppression, cardiotoxicity and other toxicities could not be assessed.

## 5 CONCLUSION

A popPK model was developed for mitoxantrone in childhood AML patients, increasing understanding of its pharmacokinetic characteristics in this patient group. Pharmacokinetics were found to be in line with previous findings from a limited number of studies in children and showed comparable CL but lower volume of distribution as compared to adults. The lower mitoxantrone exposure observed in infants administered a reduced body weight‐based dosing regimen supports the findings of previous studies calling for updated, evidence‐based chemotherapy dosing for infant patients. Simulation results suggest that removal of body weight‐based mitoxantrone dosing in younger patients will lead to more consistent drug exposures being achieved across all patient ages. Toxicity was frequently observed in the studied patient group, but its incidence was not significantly correlated with AUC, CL or dosing regimen. Ultimately, this study provides novel information relating to the pharmacokinetics of mitoxantrone in children and may aid future dosing decisions made for patients.

## AUTHOR CONTRIBUTIONS

All authors made substantial contributions to the conception/design of the research and/or the acquisition of data. AMB and HH‐S performed the primary data analysis. All authors contributed to the interpretation of findings nad/or manuscript preparation and review. All authors have given approval for the final version to be published.

## CONFLICT OF INTEREST STATEMENT

The authors declare no conflicts of interest.

## Supporting information


**Figure S1.** Correlation of patient covariates.
**Figure S2.** Prediction‐corrected visual predictive check plots.
**Table S1.** Incidence of severe (grade 3–4) toxicity in the studied patient cohort (n = 44 patients).
**Table S2.** Statistical comparison of frequently observed (>20% of patients) severe myelosuppression‐related toxicities between dosing groups (mg/kg vs mg/m^2^).
**Table S3.** Statistical comparison of frequently observed (>20% of patients) severe myelosuppression‐related toxicities between patients who were or were not co‐administered cytarabine.
**Figure S3.** Comparison of mitoxantrone area under the plasma concentration‐time curve (AUC) with the incidence of the most frequently observed (>20% of patients) severe myelosuppression‐related toxicities.
**Figure S4.** Comparison of individual mitoxantrone clearance (CL) rates with the incidence of the most frequently observed (>20% of patients) severe myelosuppression‐related toxicities.
**Figure S5.** Comparison of patient age with the incidence of the most frequently observed (>20% of patients) severe myelosuppression‐related toxicities.
**Figure S6.** Comparison of patient body weight with the incidence of the most frequently observed (>20% of patients) severe myelosuppression‐related toxicities.

## Data Availability

The data that support the findings of this study are available on request from the corresponding author.
