# Supplementary material for: Population pharmacokinetics and dose–response relationships of mitoxantrone in children with acute myeloid leukaemia
Source: Br J Clin Pharmacol. 2026 Jan 14;92(6):1760–70. doi: 10.1002/bcp.70436 (PMC13206311; doi:10.1002/bcp.70436)

**SUPPLEMENTARY INFORMATION**

**Population pharmacokinetics and dose-response relationships of mitoxantrone in children with acute myeloid leukaemia**

Andrew M Brandon^1^, Hinke Huisman-Siebinga^2^, Shelby Barnett^1^, Paul Wetherell^3^, Pamela Kearns^3^, Brenda Gibson^4^, Nicholas Heaney^4^, Owen Smith^5^, André Baruchel^6^, Arnaud Petit^7^, Andrew Moore^8^, Kayode Ogungbenro^9^, Alwin D R Huitema^2,10,11^, Gareth J Veal^1*^

^1^ Newcastle University Centre for Cancer, Translational & Clinical Research Institute, Newcastle University, Newcastle upon Tyne, UK; ^2^ Department of Pharmacy and Pharmacology, The Netherlands Cancer Institute, Amsterdam, The Netherlands; ^3^ Cancer Research UK Clinical Trials Unit, School of Medical Sciences, University of Birmingham, Birmingham, UK; ^4^ Royal Hospital for Children, Glasgow, UK; ^5^ Our Lady’s Hospital for Sick Children, Dublin, Ireland; ^6^ Hôpital Robert Debré, Assistance Publique – Hôpitaux de Paris, Paris, France; ^7^ Hôpital Trousseau, Assistance Publique – Hôpitaux de Paris, Paris, France; ^8^ Queensland Children’s Hospital, Brisbane, Australia; ^9^ Centre for Applied Pharmacokinetic Research, Division of Pharmacy and Optometry, University of Manchester, Manchester, UK; ^10^ Department of Pharmacology, Princess Máxima Center for Pediatric Oncology, Utrecht, The Netherlands; ^11^ Department of Clinical Pharmacy, University Medical Center Utrecht, Utrecht University, Utrecht, The Netherlands

**Contents:**

S1. Covariate Plots

S2. Final Population Pharmacokinetic Model Code

S3. Prediction-corrected VPC

S4. Toxicity

**S1. Covariate Plots**

**Figure S1.** Correlation of patient covariates.


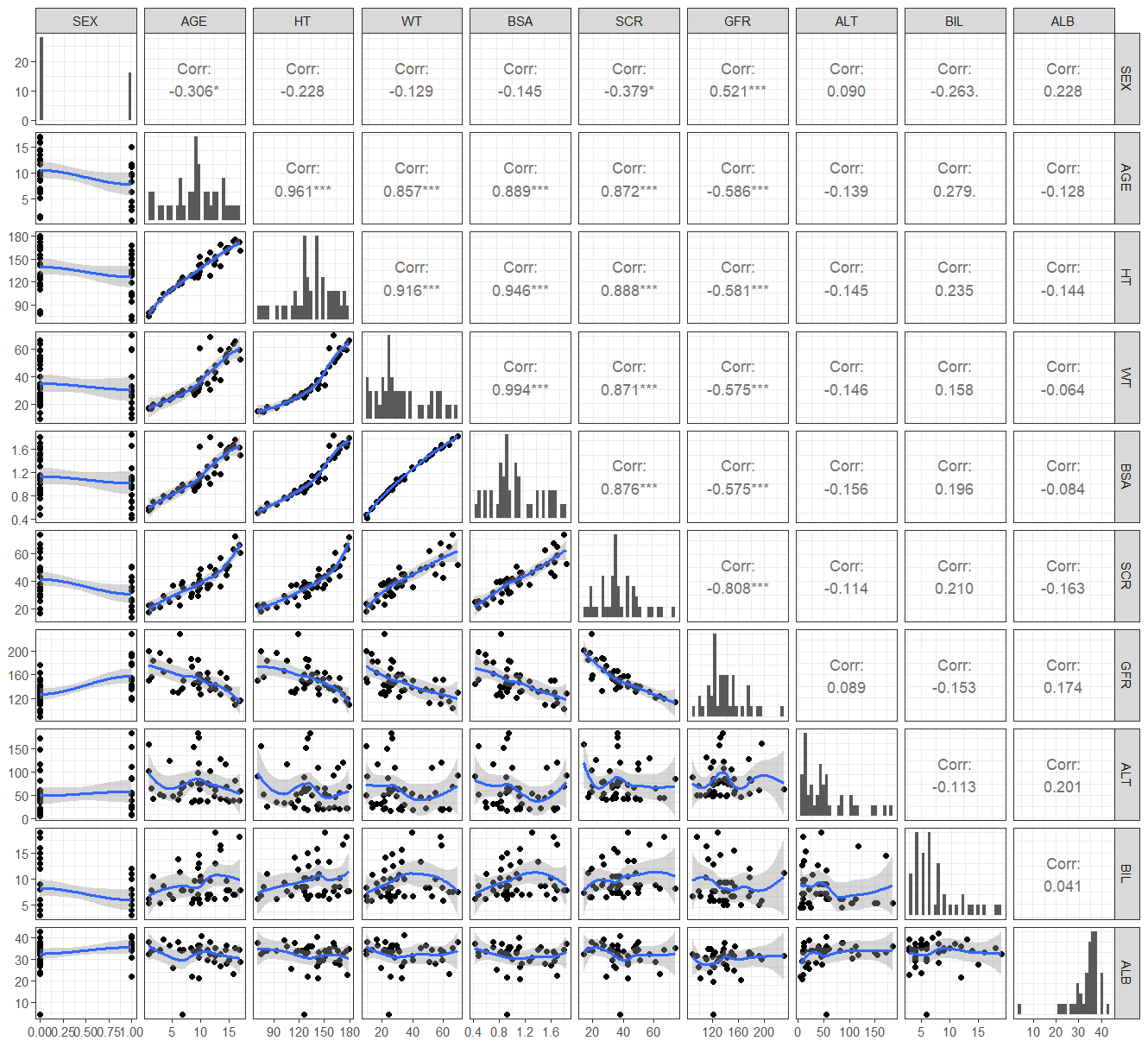


Correlation of available patient covariates produced in R using the ggplot2 and GGally packages. Where SEX = 0 is M, SEX = 1 is F; AGE is in years; HT = Height (cm); WT = Body weight (kg); BSA = Body surface area (m^2^); SCR = Serum creatinine (µmol/L); GFR = eGFR (mL/min/1.73m^2^); ALT = Alanine aminotransferase (U/L); BIL = Bilirubin (µmol/L); ALB = Serum albumin (g/L).

**S2. Final Population Pharmacokinetic Model Code**

$PROBLEM mitoxantrone
;----------------------------------
$INPUT ID AMT RATE TIME DV SEX AGE HT WT BSA SCR GFR ALT BIL ALB DOSEREG LINE COURSE EVID MDV OUTL BLQ
;----------------------------------
$DATA "../../data/Mitoxantrone_tidied_nonmem.csv" ACCEPT(OUTL.EQ.0)
;----------------------------------
$SUBROUTINES ADVAN3 TRANS4
;----------------------------------
$PK ; MU referencing for SAEM
LTVCL = LOG(THETA(1)) + THETA(5)*LOG(WT/27.5)
TVV1 = THETA(2) * (WT/27.5)**THETA(6)
TVQ = THETA(3) * (WT/27.5)**THETA(5)
LTVV2 = LOG(THETA(4)) + THETA(6)*LOG(WT/27.5)

MU_1 = LTVCL
MU_2 = LTVV2

CL = EXP(MU_1 + ETA(1))
V1 = TVV1
Q = TVQ
V2 = EXP(MU_2 + ETA(2))

S1 = V1/1000 ; scale concentration units
;----------------------------------
$THETA
39.1 ; CL ; L/h ;(1)
23.2 FIX ; V1 ; L ;(2)
27.6 ; Q ; L/h ;(3)
85.9 ; V2 ; L ;(4)

0.75 FIX ; WTCLQ ;;(5)
1 FIX ; WTV ;;(6)

(0, 0.382) ; PROP ERR ;;(7)
2.5 FIX ; ADD ERR ;;(8) ; 1/2 LOQ

$OMEGA BLOCK(2)
0.344 ; IIV CL ;;(ETA1)
-0.426 1.02 ; IIV V2 ;;(ETA2)

$SIGMA
1 FIX
;----------------------------------
$ERROR
LOQ = 5 ;ng/mL

W = 1
IPRED = F
IF(F.NE.0) W = SQRT((THETA(7)*IPRED)**2+THETA(8)**2)

DUM = (LOQ-IPRED)/W
CUMD = PHI(DUM)+1E-10

IF(BLQ.EQ.0.OR.NPDE_MODE.EQ.1) THEN
F_FLAG = 0
Y = IPRED + W*EPS(1)
ENDIF

IF(BLQ.EQ.1.AND.NPDE_MODE.EQ.0) THEN
F_FLAG = 1
Y = CUMD
MDVRES = 1
ENDIF

IF(BLQ.EQ.1) DV_LOQ = LOQ
;----------------------------------
; Include time after last dose column
IF(NEWIND.LT.2) THEN
IFL=0
TAD=0.0
ENDIF

IF(EVID.EQ.1.OR.EVID.EQ.4) THEN
TDOS=TIME
TAD=0.0
IFL=1
ENDIF

IF(IFL.EQ.1.AND.EVID.NE.1.AND.EVID.NE.4)TAD=TIME-TDOS
;----------------------------------
$EST METHOD=SAEM INTERACTION NBURN=2000 NITER=2000 PRINT=100 CTYPE=3 LAPLACIAN NUMERICAL SLOW
$EST METHOD=IMP EONLY=1 NITER=20 ISAMPLE=1000 MAPITER=0 PRINT=1
;----------------------------------
$COV PRINT=E
;----------------------------------
$TABLE ID TIME LINE COURSE DV MDV EVID PRED IPRED TAD CWRES NPDE NPD ONEHEADER NOPRINT FILE=sdtab1 NPDTYPE=1 ESAMPLE=1000 SEED=12345
$TABLE ID CL V1 Q V2 ETAS(1:LAST) ONEHEADER NOPRINT FILE=patab1 ; model parameters
$TABLE ID SEX DOSEREG ONEHEADER NOPRINT FILE=catab1 ; categorical covariates
$TABLE ID AGE HT WT BSA SCR GFR ALT BIL ALB ONEHEADER NOPRINT FILE=cotab1 ; continuous covariates
;full table for R plots
$TABLE ID CL V1 Q V2 ETAS(1:LAST) TIME DV MDV EVID SEX AGE HT WT BSA SCR GFR ALT BIL ALB AMT DOSEREG LINE COURSE TAD CUMD PRED IPRED CWRES NPDE NPD NOTITLE ONEHEADER NOPRINT FILE=fulltab.tab

**S3. Prediction-corrected VPC**

**Figure S2.** Prediction-corrected visual predictive check plots.


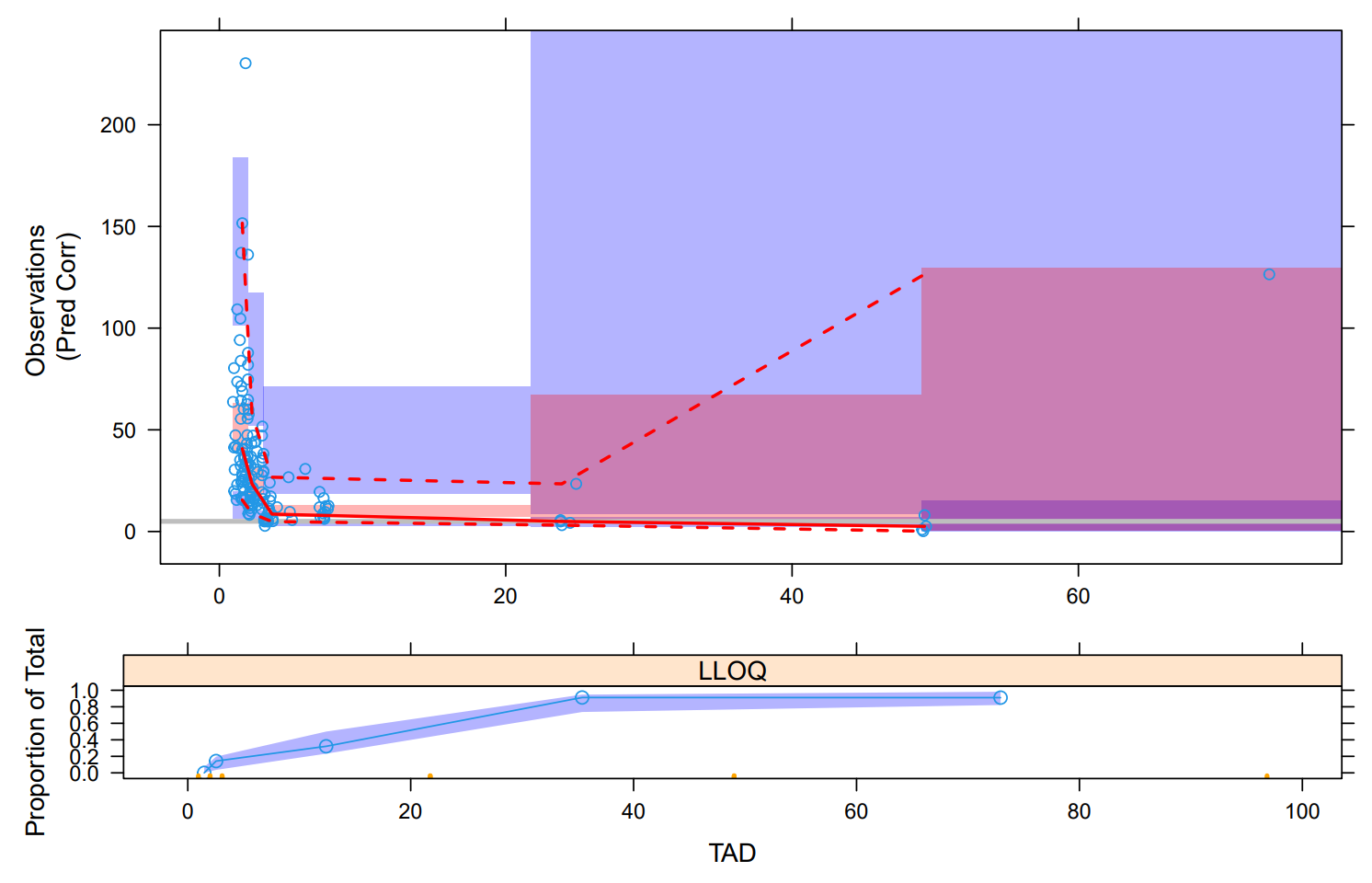


PsN generated VPC plots including both prediction correction (*-predcorr*) and filtering of BLLOQ data (*-lloq*). Note that specifying the LLOQ in the VPC is required due to the use of the M3 method and the large proportion of BLLOQ data. However, prediction correction is calculated using all simulated values, prior to censoring of data. Censoring is then applied, followed by the correction. As a result, values originally treated as censored data can appear outside the LLOQ boundary in the final plot.

**S4. Toxicity**

**Table S1.** Incidence of severe (grade 3-4) toxicity in the studied patient cohort (n = 44 patients).

| **Toxicity** | **Toxicity Count** | **Frequency (%)** |
| --- | --- | --- |
| Febrile neutropenia | 12 | 27.3 |
| Platelet count decreased | 12 | 27.3 |
| Neutrophil count decreased | 11 | 25.0 |
| White blood cell decreased | 10 | 22.7 |
| Lymphocyte count decreased | 10 | 22.7 |
| Anaemia | 10 | 22.7 |
| Hypokalemia | 3 | 6.8 |
| Device related infection | 3 | 6.8 |
| Hyperkalemia | 2 | 4.5 |
| Rash maculo-papular | 2 | 4.5 |
| Infections and infestations - Other | 2 | 4.5 |
| Pleural effusion | 2 | 4.5 |
| Sepsis | 2 | 4.5 |
| Hypotension | 2 | 4.5 |
| Upper respiratory infection | 2 | 4.5 |
| Fever | 2 | 4.5 |
| Hyponatremia | 2 | 4.5 |
| Hypoxia | 2 | 4.5 |
| Nausea | 1 | 2.3 |
| Pulmonary edema | 1 | 2.3 |
| Ascites | 1 | 2.3 |
| Supraventricular tachycardia | 1 | 2.3 |
| Sinus tachycardia | 1 | 2.3 |
| Soft tissue infection | 1 | 2.3 |
| Hypophosphatemia | 1 | 2.3 |
| Arthralgia | 1 | 2.3 |
| Hoarseness | 1 | 2.3 |
| Mucositis oral | 1 | 2.3 |
| Lung infection | 1 | 2.3 |
| Lymph gland infection | 1 | 2.3 |
| Anorexia | 1 | 2.3 |
| Blood bilirubin increased | 1 | 2.3 |
| Anal pain | 1 | 2.3 |
| Anorectal infection | 1 | 2.3 |
| Lip infection | 1 | 2.3 |
| Epistaxis | 1 | 2.3 |
| Leukocytosis | 1 | 2.3 |
| Lymphocyte count increased | 1 | 2.3 |
| Hypernatremia | 1 | 2.3 |
| Hypoalbuminemia | 1 | 2.3 |
| Dizziness | 1 | 2.3 |
| Headache | 1 | 2.3 |
| Pleural hemorrhage | 1 | 2.3 |
| Respiratory, thoracic and mediastinal disorders - Other | 1 | 2.3 |
| Hematuria | 1 | 2.3 |
| Renal and urinary disorders - Other | 1 | 2.3 |
| Alanine aminotransferase increased | 1 | 2.3 |

**Table S2.** Statistical comparison of frequently observed (>20% of patients) severe myelosuppression-related toxicities between dosing groups (mg/kg vs mg/m^2^).

| **Toxicity** | **p-value** |
| --- | --- |
| Febrile neutropenia | 0.1238 |
| Platelet count decreased | 0.1238 |
| Neutrophil count decreased | 0.0938 |
| White blood cell decreased | 0.0676 |
| Lymphocyte count decreased | 0.0676 |
| Anaemia | 0.0676 |

Using the Mann-Whitney U Test (Wilcoxon Rank Sum Test), with p<0.05 considered significant.

**Table S3.** Statistical comparison of frequently observed (>20% of patients) severe myelosuppression-related toxicities between patients who were or were not co-administered cytarabine.

| **Toxicity** | **p-value** |
| --- | --- |
| Febrile neutropenia | 0.2616 |
| Platelet count decreased | 0.2616 |
| Neutrophil count decreased | 0.1572 |
| White blood cell decreased | 0.0835 |
| Lymphocyte count decreased | 0.0835 |
| Anaemia | 0.8010 |

Using the Mann-Whitney U Test (Wilcoxon Rank Sum Test), with p<0.05 considered significant.

**Figure S3.** Comparison of mitoxantrone area under the plasma concentration-time curve (AUC) with the incidence of the most frequently observed (>20% of patients) severe myelosuppression-related toxicities.


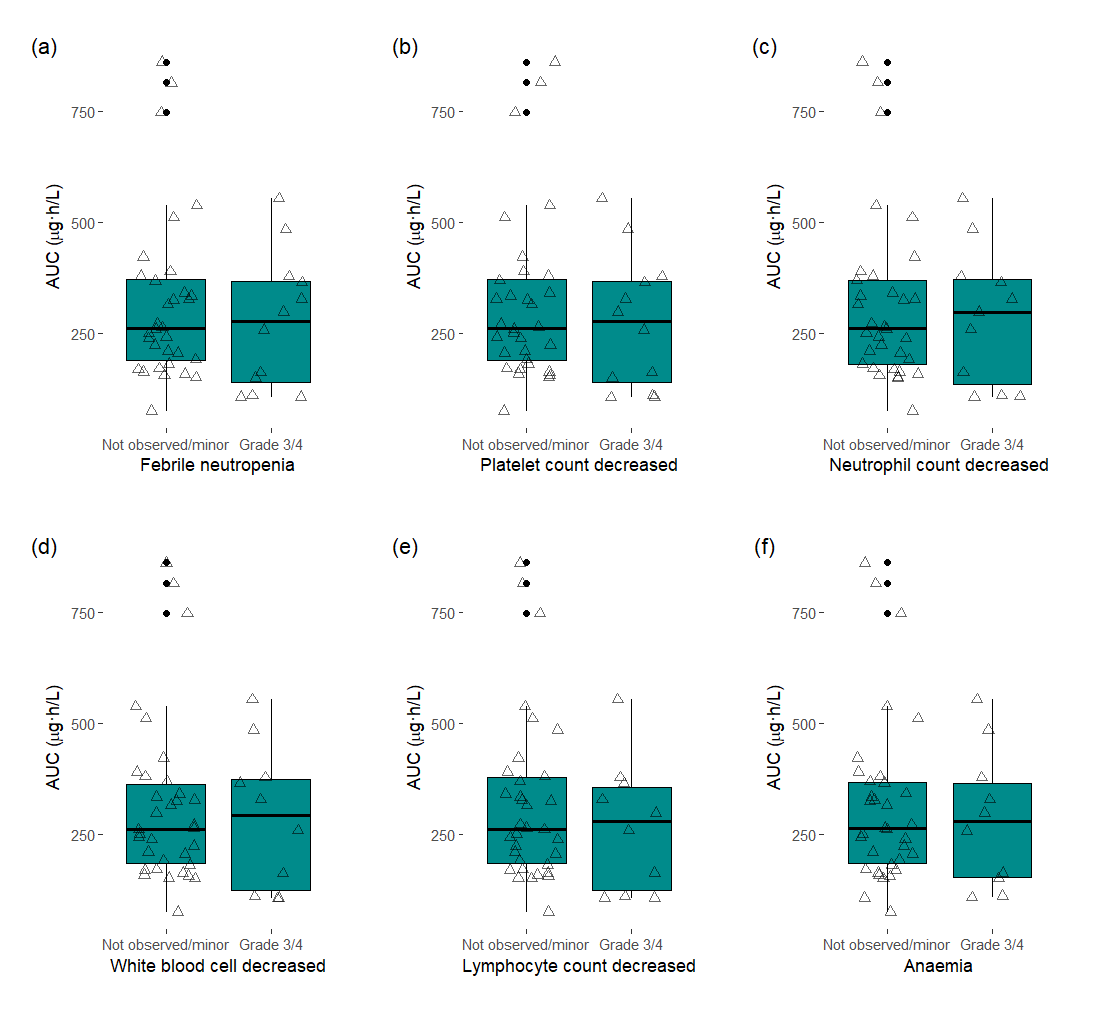


**Figure S4.** Comparison of individual mitoxantrone clearance (CL) rates with the incidence of the most frequently observed (>20% of patients) severe myelosuppression-related toxicities.


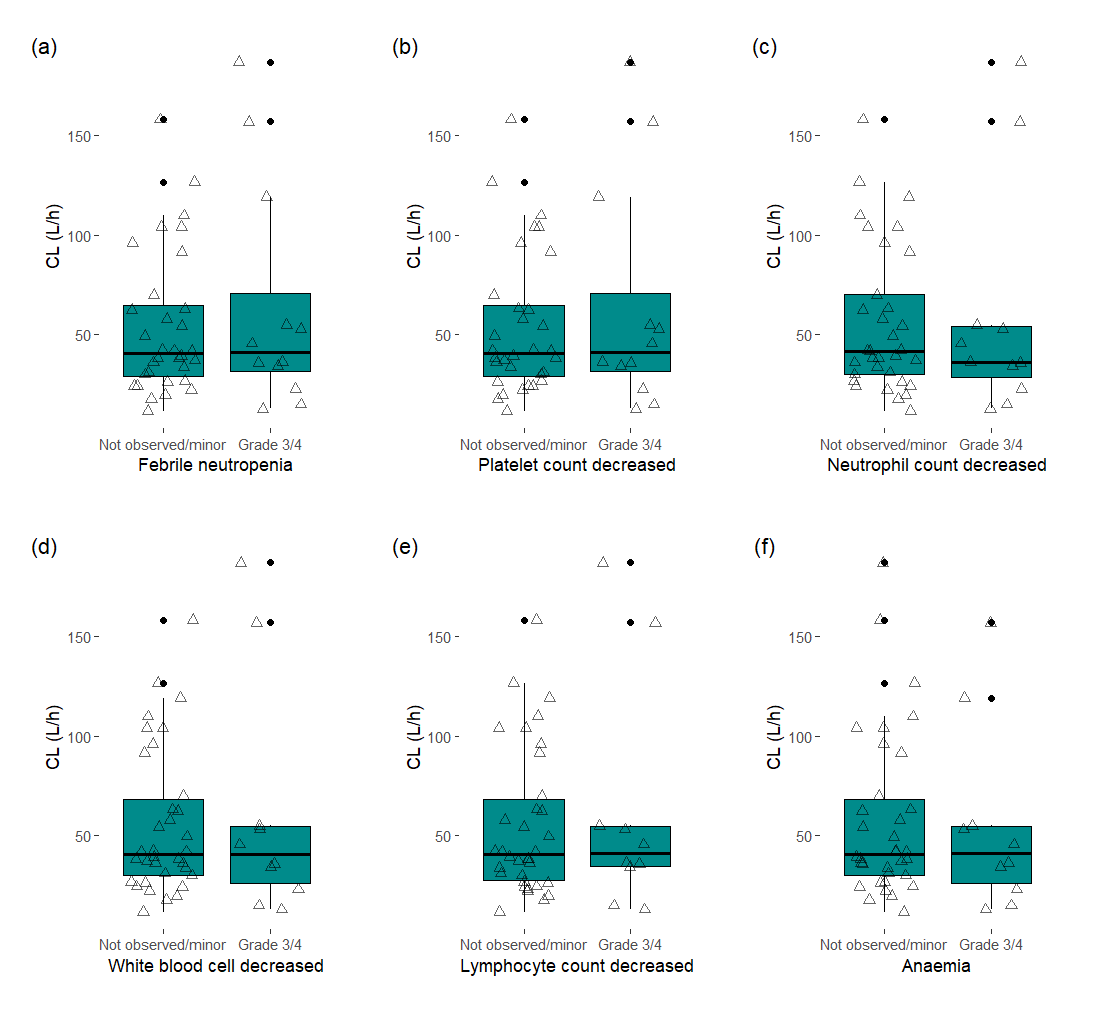


**Figure S5.** Comparison of patient age with the incidence of the most frequently observed (>20% of patients) severe myelosuppression-related toxicities.


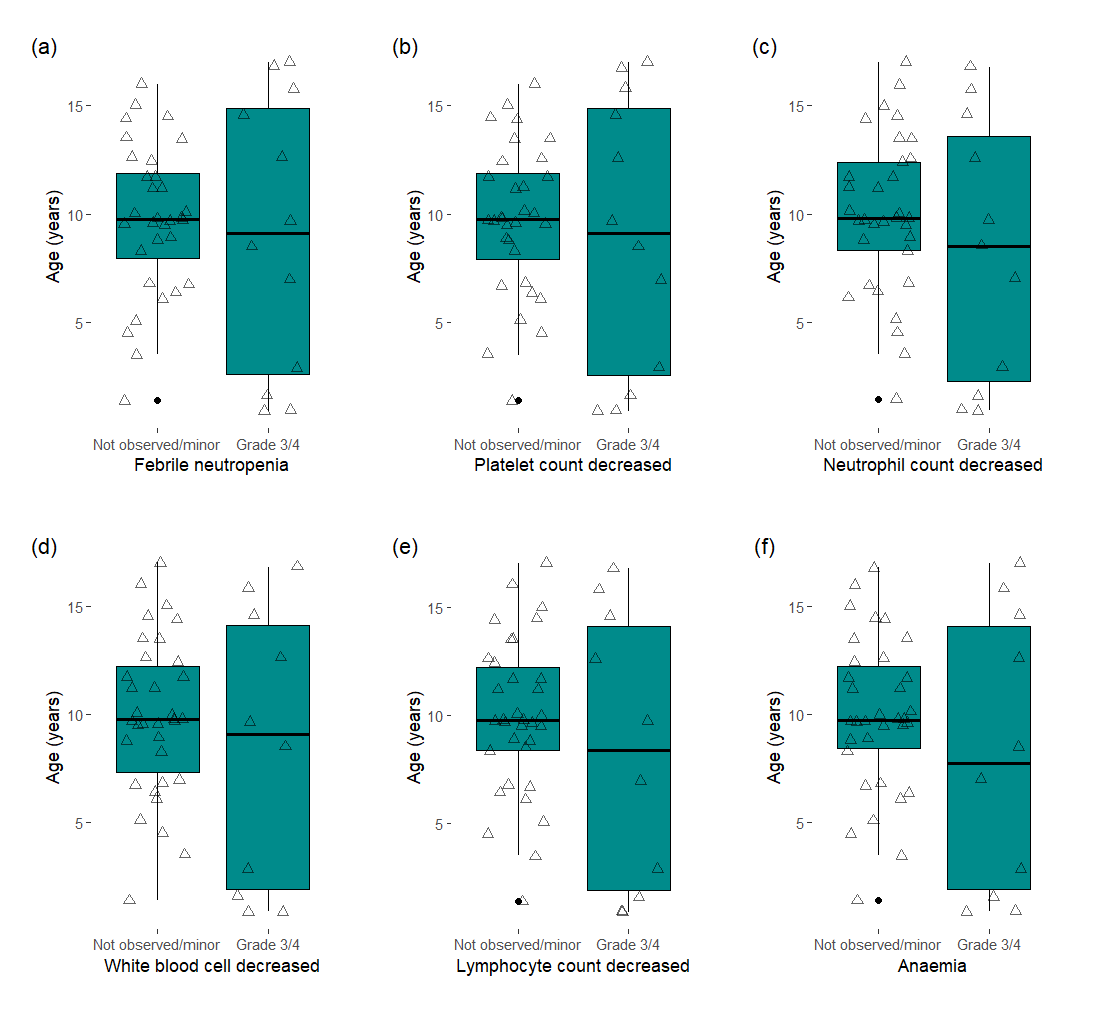


**Figure S6.** Comparison of patient body weight with the incidence of the most frequently observed (>20% of patients) severe myelosuppression-related toxicities.


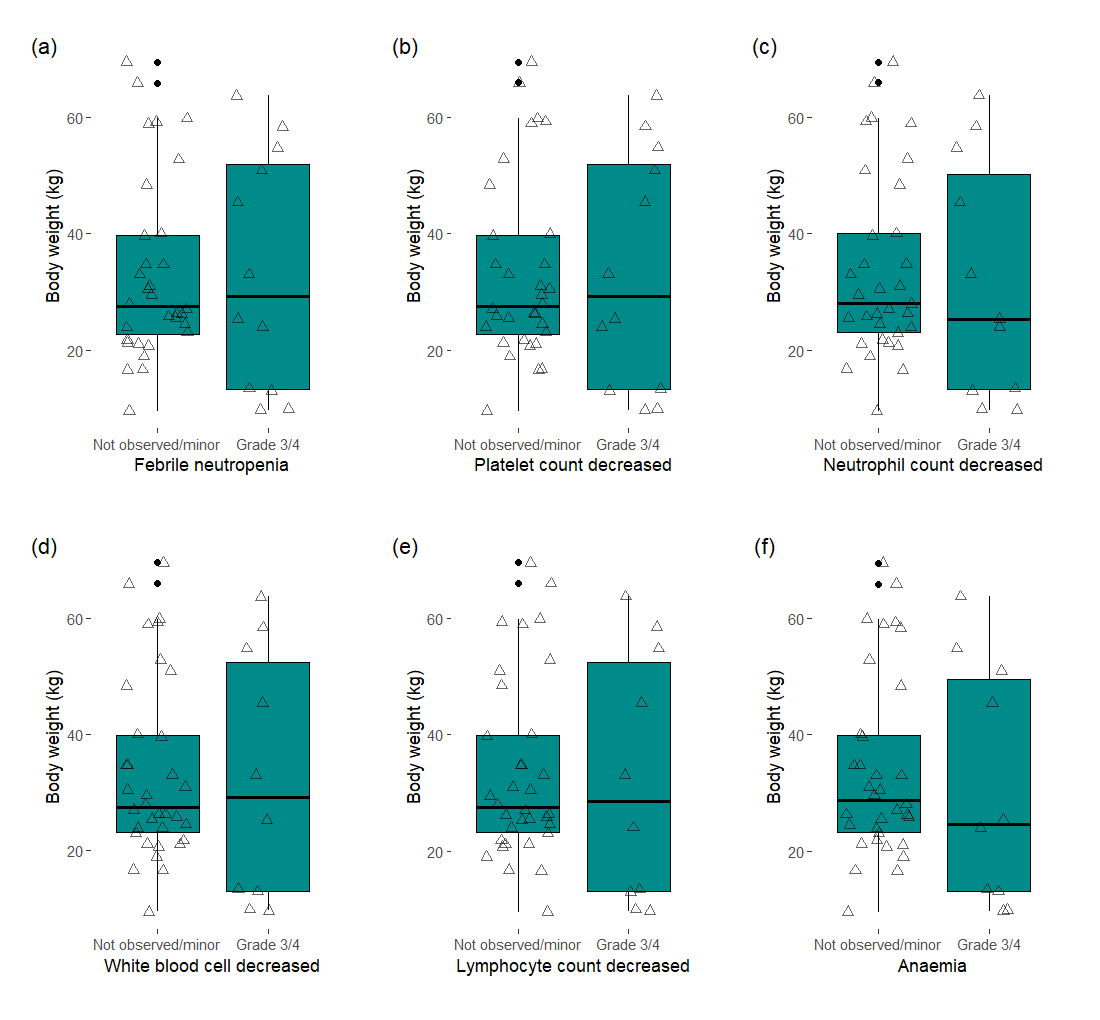

Supplement: Supplementary file 1 — Figure S1. Correlation of patient covariates. Figure S2. Prediction‐corrected visual predictive check plots. Table S1. Incidence of severe (grade 3–4) toxicity in the studied patient cohort (n = 44 patients). Table S2. Statistical comparison of frequently observed (>20% of patients) severe myelosuppression‐related toxicities between dosing groups (mg/kg vs mg/m2). Table S3. Statistical comparison of frequently observed (>20% of patients) severe myelosuppression‐related toxicities between patients who were or were not co‐administered cytarabine. Figure S3. Comparison of mitoxantrone area under the plasma concentration‐time curve (AUC) with the incidence of the most frequently observed (>20% of patients) severe myelosuppression‐related toxicities. Figure S4. Comparison of individual mitoxantrone clearance (CL) rates with the incidence of the most frequently observed (>20% of patients) severe myelosuppression‐related toxicities. Figure S5. Comparison of patient age with the incidence of the most frequently observed (>20% of patients) severe myelosuppression‐related toxicities. Figure S6. Comparison of patient body weight with the incidence of the most frequently observed (>20% of patients) severe myelosuppression‐related toxicities. [file BCP-92-1760-s001.docx]
